# Supplementary material for: Commercial milk formula feeding among children under two years in Nepal: Trends and determinants from four Nepal Demographic and Health Surveys (2006–2022)
Source: PLoS One. 2026 Jan 2;21(1):e0339128. doi: 10.1371/journal.pone.0339128 (PMC12758697; doi:10.1371/journal.pone.0339128)
Supplement: S1 Table — Provides details on all potential variables included in the analysis and their categorisation into enabling and underlying factors. (DOCX) [file pone.0339128.s001.docx]

**S1 Table. Identification and categorisation of potential variables used in the study**

| **Variables** | **Identification** | **Reference category** |
| --- | --- | --- |
| **NDHS year** | 1. 2006 2. 2011 3. 2016 4. 2022 | Year 2006 |
| **Enabling Factors** |  |  |
| **Place of residence** | 1. Urban 2. Rural | Staying in rural area |
| **Ecological zone** | 1. Mountain 2. Hill 3. Terai | Mountain |
| **Province** | 1. Koshi 2. Madhesh 3. Bagmati 4. Gandaki 5. Lumbini 6. Karnali 7. Sudurpaschim | Koshi |
| **Underlying factors** |  |  |
| **Infant characteristics** |  |  |
| **Child sex** | 1. Male 2. Female | Female child |
| **Perceived size at birth** | 0. Small 1. Average 2. Large | Large |
| **Preceding birth interval** | 0. No previous birth 1. <24 months 2. ≥ 24 months | No previous birth |
| **Initiation of breastfeeding** | 0. More than one hour 1. Immediately | More than an hour |
| **Obstetric and health service related characteristics** |  |  |
| **Assistance during child-birth** | 1. Health personnel 2. Relative/TBA/Others 3. No one | Relative/TBA/Others |
| **PNC check within two days for mother** | 0. No 1. Yes | No |
| **Caesarean section birth** | 0. No 1. Yes | No |
| **Place of child birth** | 0. Elsewhere 1. Health facility | Elsewhere |
| **Antenatal visits** | 0. <4 visits 1. ≥ 4 visits | <4 visits |
| **Sociodemographic and household characteristics** |  |  |
| **Maternal age (years)** | 1. <24 2. 25-34 3. 35-49 years | <24 years |
| **Caste/Ethnicity** | 0. Brahmin/Chhetri 1. Madheshi 2. Dalit 3. Janajati 4. Muslim | Janajati |
| **Maternal employment status** | 0. Currently not working 1. Currently working | Currently not working |
| **Paternal employment status** | 0. Currently not working 1. Currently working | Currently not working |
| **Wealth index** | 1. Poorest 2. Poorer 3. Middle 4. Richer 5. Richest | Poorest |
| **Media exposure** | 0. Not at all 1. Less than once a week 2. Atleast once a week | Not al all |
| **Household size** | 1. 1-3 2. 4-5 3. 6-38 | .6-38 |
| **Maternal education** | 0. No education 1. Primary education 2. Secondary and higher | No education |
| **Paternal education** | 0. No education 1. Primary education 2. Secondary and higher | No education |
