# Supplementary material for: Commercial milk formula feeding among children under two years in Nepal: Trends and determinants from four Nepal Demographic and Health Surveys (2006–2022)
Source: PLoS One. 2026 Jan 2;21(1):e0339128. doi: 10.1371/journal.pone.0339128 (PMC12758697; doi:10.1371/journal.pone.0339128)
Supplement: S2 Table — Presents the results of multivariable binary logistic regression analyses assessing determinants of commercial milk formula feeding. (DOCX) [file pone.0339128.s002.docx]

**S2 Table. Association of enabling and underlying factors with commercial milk formula feeding practices among children aged 0-5 months and 6-23 months based on pooled data analyses 2006-2022 in Nepal, 2022: results from multivariable binary logistic regression analysis**

**Full results of regression model**

| Variables | 0-5 months | | 6-23 months | |
| --- | --- | --- | --- | --- |
|  | unadjusted OR (95% CI) | adjusted OR (95% CI) | unadjusted OR (95% CI) | adjusted OR (95% CI) |
| Survey year |  |  |  |  |
| NDHS 2006 | 1.00 | 1.00 | 1.00 | 1.00 |
| NDHS 2011 | 0.57 (0.18, 1.77) | 0.73 (0.18, 2.93) | 1.13 (0.59, 2.15) | 0.86 (0.39, 1.90) |
| NDHS 2016 | 2.37 (0.83, 6.78) | 1.50 (0.33, 6.86) | 1.26 (0.68, 2.31) | 0.60 (0.26, 1.41) |
| NDHS 2022 | 6.26 (2.45, 16.00) *** | 5.40 (1.41, 20.77) ** | 3.44 (1.99, 5.94) *** | 1.68 (0.80, 3.66) |
| Enabling Factors |  |  |  |  |
| Place of residence |  |  |  |  |
| Urban | 8.06 (4.38, 14.82) *** | 1.61 (0.70, 3.72) | 2.87 (1.99, 4.15) *** | 0.94 (0.61, 1.44) |
| Rural | 1.00 | 1.00 | 1.00 | 1.00 |
| Province |  |  |  |  |
| Koshi | 1.00 | 1.00 | 1.00 | 1.00 |
| Madhesh | 0.20 (0.06, 0.65) ** | 0.35 (0.08, 1.48) | 0.88 (0.47, 1.66) | 1.20 (0.55, 2.65) |
| Bagmati | 2.11 (1.06, 4.20) * | 1.50 (0.71, 3.19) | 2.47 (1.38, 4.43) ** | 1.75 (0.99, 3.11) |
| Gandaki | 0.65 (0.27, 1.58) | 0.63 (0.23, 1.68) | 1.08 (0.52, 2.24) | 1.11 (0.52, 2.34) |
| Lumbini | 0.64 (0.24, 1.65) | 0.68 (0.26, 1.79) | 1.09 (0.59, 2.01) | 1.04 (0.54, 2.00) |
| Karnali | 0.04 (0.00, 0.29) ** | 0.04 (0.01, 0.39) ** | 0.62 (0.29, 1.35) | 1.10 (0.49, 2.46) |
| Sudurpaschim | 0.05 (0.01, 0.26) *** | 0.06 (0.01, 0.33) ** | 1.00 (0.45, 2.21) | 1.34 (0.57, 3.11) |
| Underlying factors | | | | |
| Infant characteristics |  |  |  |  |
| Child sex |  |  |  |  |
| Male | 0.98 (0.55, 1.75) | 1.13 (0.58, 2.20) | 1.74 (1.22, 2.49) ** | 1.67 (1.15, 2.42) ** |
| Female | 1.00 | 1.00 | 1.00 | 1.00 |
| Perceived size at birth |  |  |  |  |
| Small |  |  | 2.02 (1.15, 3.55) * | 2.76 ( 1.48, 5.15) ** |
| Average |  |  | 1.40 (0.82, 2.37) | 1.31 (0.74, 2.32) |
| Large |  |  | 1.00 | 1.00 |
| Preceding birth interval |  |  |  |  |
| No previous birth | 12.04 (1.62, 89.34) * | 3.55 (0.39, 32.09) | 6.23 (2.53, 15.37) *** | 2.59 (1.02, 6.56) * |
| <24 months | 1.00 | 1.00 | 1.00 | 1.00 |
| >=24 months | 8.49 (1.14, 63.14) * | 3.00 (0.35, 26.06) | 4.01 (1.59, 10.10) ** | 2.51 (0.99, 6.37) |
| Initiation of breastfeeding |  |  |  |  |
| More than 1 hour | 1.00 | 1.00 |  |  |
| Immediately | 0.66 (0.38, 1.15) | 1.13 (0.57, 2.25) |  |  |
| Obstetric and health service-related characteristics | | | | |
| Provider of Delivery During Labour |  |  |  |  |
| Health personnel | 1.00 | 1.00 | 1.00 | 1.00 |
| TBA/Relative/Others | 0.15 (0.07, 0.36) *** | 0.71 (0.17, 3.04) | 0.26 (0.17, 0.41) *** | 1.45 (0.67, 3.11) |
| No One | 0.18 (0.02, 1.31) | 0.71 (0.05, 8.70) | 0.09 (0.02, 0.45) ** | 1.25 (0.20, 7.87) |
| PNC check within two days |  |  |  |  |
| No | 1.00 | 1.00 | 1.00 | 1.00 |
| Yes | 5.67 (3.06, 10.50) *** | 1.02 (0.34, 3.09) | 3.43 (2.37, 4.99) *** | 1.26 (0.73, 2.18) |
| Delivery by caesarean section |  |  |  |  |
| No | 1.00 | 1.00 | 1.00 | 1.00 |
| Yes | 7.53 (4.13, 13.74) *** | 2.16 (1.01, 4.59) | 4.75 (3.24, 6.95) *** | 1.36 (0.90, 2.06) |
| Place of child birth |  |  |  |  |
| Elsewhere | 1.00 | 1.00 | 1.00 | 1.00 |
| Health facilities | 9.63 (4.27, 21.71) | 0.68 (0.11, 4.14) | 6.87 (4.21, 11.20) *** | 3.18 (1.16, 8.73) * |
| Antenatal visits |  |  |  |  |
| <4 ANC visits | 1.00 | 1.00 |  |  |
| >=4 ANC visits | 4.58 (2.27, 9.24) | 1.17 (0.41, 3.38) |  |  |
| Sociodemographic and household characteristics | | | | |
| Maternal age (years) |  |  |  |  |
| <24 | 1.00 | 1.00 | 1.00 | 1.00 |
| 25-34 | 3.56 (2.03, 6.24) *** | 2.29 (1.14, 4.60) * | 1.64 (1.14, 2.35) ** | 1.29 (0.89, 1.86) |
| 35-49 | 1.84 (0.56, 6.03) | 3.41 (0.58, 20.07) | 0.83 (0.38, 1.84) | 1.04 (0.43, 2.53) |
| Caste/Ethnicity |  |  |  |  |
| Brahmin/Chhetri | 1.27 (0.67, 2.41) | 1.02 (0.46, 2.27) | 1.60 (1.05, 2.44) * | 1.01 (0.64, 1.59) |
| Madheshi | 0.43 (0.18, 1.03) | 0.87 (0.22, 3.52) | 0.90 (0.52, 1.57) | 1.05 (0.54, 2.04) |
| Dalit | 0.34 (0.12, 0.92) * | 1.77 (0.55, 65.70) | 0.43 (0.21, 0.87) * | 0.69 (0.34, 1.37) |
| Janajati | 1.00 | 1.00 | 1.00 | 1.00 |
| Muslim | 0.71 (0.25, 2.02) | 2.51 (0.49, 12.87) | 0.54 (0.21, 1.37) | 0.90 (0.30, 2.71) |
| Maternal employment status |  |  |  |  |
| Currently not working |  |  | 1.00 | 1.00 |
| Currently Working |  |  | 0.68 (0.47, 0.96) * | 1.03 (0.69, 1.54) |
| Wealth index |  |  |  |  |
| Poorest | 1.00 | 1.00 | 1.00 | 1.00 |
| Poorer | 0.89 (0.24, 3.39) | 0.42 (0.09, 1.98) | 3.69 (1.64, 8.30) ** | 3.08 (1.35, 7.01) ** |
| Middle | 0.60 (0.15, 2.35) | **0.17 (0.03, 0.84) *** | 3.90 (1.86, 8.14) *** | 3.02 (1.40, 6.51) ** |
| Richer | 5.95 (2.17, 16.28) *** | 1.63 (0.51, 5.25) | 5.52 (2.69, 11.33) *** | 3.01 (1.31, 6.92)** |
| Richest | 19.46 (7.49, 50.58) *** | 2.13 (0.55, 8.30) | 18.48 (9.48, 36.00) *** | 5.87 (2.42, 14.22)*** |
| Media exposure |  |  |  |  |
| Not at all | 1.00 | 1.00 | 1.00 | 1.00 |
| Less than once a week | 1.50 (0.63, 3.55) | 0.57 (0.21, 1.52) | 1.10 (0.62, 1.97) | 0.76 (0.42, 1.39) |
| At least once a week | 2.58 (1.23, 5.40) * | 0.53 (0.22, 1.30) | 2.39 (1.44, 3.95) *** | 1.01 (0.59, 1.73) |
| Household size |  |  |  |  |
| 1-3 | 2.82 (1.18, 6.73) * | 2.43 (0.91, 6.54) |  |  |
| 4-5 | 1.29 (0.70, 2.35) | 0.84 (0.38, 1.88) |  |  |
| 6-38 | 1.00 | 1.00 |  |  |
| Maternal education |  |  |  |  |
| No education | 1.00 | 1.00 | 1.00 | 1.00 |
| Primary | 2.26 (0.48, 10.74) | 1.39 (0.24, 8.10) | 1.70 (0.93, 3.10) | 0.79 (0.38, 1.65) |
| Secondary and higher | 26.61 (7.51, 94.25) *** | 12.48 (2.63, 59.14) ** | 5.79 (3.44, 9.74) *** | 1.50 (0.73, 3.08) |
| Paternal education |  |  |  |  |
| No education | 1.00 | 1.00 |  | 1.00 |
| Primary | 5.42 (1.52, 19.33) ** | 0.69 (0.11, 4.38) | 4.84 (2.15, 10.88) *** | 2.22 (0.93, 5.33) |
| Secondary and higher | 19.21 (5.05, 73.04) *** | 0.96 (0.14, 6.48) | 16.42 (7.07, 38.13) *** | 3.14 (1.22, 8.10) * |

***Significant at p-value < 0.001. **Significant at p-value < 0.01. *Significant at p-value < 0.05; 1.00 represents the reference category.
