# Supplementary material for: Commercial milk formula feeding among children under two years in Nepal: Trends and determinants from four Nepal Demographic and Health Surveys (2006–2022)
Source: PLoS One. 2026 Jan 2;21(1):e0339128. doi: 10.1371/journal.pone.0339128 (PMC12758697; doi:10.1371/journal.pone.0339128)
Supplement: S3 Table — Shows robustness checks using individual survey subsets for infants aged 0–5 months. (DOCX) [file pone.0339128.s003.docx]

**S3 Table. Sensitivity analysis of the pooled analysis on subsets of data (NDHS 2022, 2016, 2011, & 2006) among 0-5-month infants**

| **Variables** | **Pooled (2006 - 2022)** | **2022** | **2016** | **2011** | **2006** |
| --- | --- | --- | --- | --- | --- |
|  | **adjusted OR (95% CI)** | **adjusted OR (95% CI)** | **adjusted OR (95% CI)** | **adjusted OR (95% CI)** | **adjusted OR (95% CI)** |
| **Survey year** |  |  |  |  |  |
| NDHS 2006 | 1 |  |  |  |  |
| NDHS 2011 | 0.73 (0.18, 2.93) |  |  |  |  |
| NDHS 2016 | 1.50 (0.33, 6.86) |  |  |  |  |
| NDHS 2022 | 5.40 (1.41, 20.77) ** |  |  |  |  |
| **Enabling Factors** |  |  |  |  |  |
| **Place of residence** |  |  |  |  |  |
| Urban | 1.61 (0.70, 3.72) | 0.88 (0.27, 2.88) | 1.90 (0.25, 14.30) | 18.02 (3.12, 104.07) | 3.37 (0.52, 21.93) |
| Rural | 1 | 1 | 1 | 1 | 1 |
| **Province** |  |  |  |  |  |
| Koshi | 1 | 1 | 1 | 1 | 1 |
| Madhesh | 0.35 (0.08, 1.48) | 0.77 (0.09, 6.65) | 0.16 (0.01, 3.55) |  |  |
| Bagmati | 1.50 (0.71, 3.19) | 3.02 (1.16, 7.85) * | 0.49 (0.08, 2.99) | 0.95 (0.14, 6.28) | 1.30 (0.10, 16.97) |
| Gandaki | 0.63 (0.23, 1.68) | 0.72 (0.20, 2.67) | 0.62 (0.12, 3.22) | 2.17 (0.20, 23.91) |  |
| Lumbini | 0.68 (0.26, 1.79) | 0.88 (0.18, 4.22) | 0.09 (0.01, 1.13) | 3.21 (0.46, 22.44) |  |
| Karnali | 0.04 (0.01, 0.39) ** | 0.06 (0.01, 0.67) * |  |  |  |
| Sudurpaschim | 0.06 (0.01, 0.33) ** | 0.06 (0.01, 0.63) * |  | 0.56 (0.05, 6.23) |  |
| **Underlying factors** | | | |  |  |
| **Infant characteristics** |  |  |  |  |  |
| **Child sex** |  |  |  |  |  |
| Male | 1.13 (0.58, 2.20) | 1.47 (0.58, 3.72) | 1.56 (0.32, 7.66) | 0.47 (0.11, 1.99) | 0.20 (0.02, 1.67) |
| Female | 1 | 1 | 1 | 1 | 1 |
| **Perceived size at birth** |  |  |  |  |  |
| Small |  |  |  |  |  |
| Average |  |  |  |  |  |
| Large |  |  |  |  |  |
| **Preceding birth interval** |  |  |  |  |  |
| No previous birth | 3.55 (0.39, 32.09) | 1.37 (0.55, 3.42) | 1.66 (0.14, 19.03) |  |  |
| <24 months | 1 | 1 | 1 |  |  |
| >=24 months | 3.00 (0.35, 26.06) |  | 0.24 (0.01, 6.60) |  |  |
| **Initiation of breastfeeding** |  |  |  |  |  |
| More than 1 hour | 1 | 1 | 1 |  |  |
| Immediately | 1.13 (0.57, 2.25) | 0.37 (0.13, 1.09) | 5.02 (0.80, 31.38) |  |  |
| **Obstetric and health service-related characteristics** | | | |  |  |
| **Provider of Delivery During Labour** |  |  |  |  |  |
| Health personnel | 1 | 1 | 1 |  |  |
| TBA/Relative/Others | 0.71 (0.17, 3.04) | 0.76 (0.15, 3.97) | 0.07 (0.00, 7.51) |  |  |
| No One | 0.71 (0.05, 8.70) |  | 0.14 (0.00, 39.50) |  |  |
| **PNC check within two days** |  |  |  |  |  |
| No | 1 | 1 | 1 |  |  |
| Yes | 1.02 (0.34, 3.09) | 0.49 (0.10, 2.34) | 2.42 (0.47, 12.32) |  |  |
| **Delivery by caesarean section** |  |  |  |  |  |
| No | 1 | 1 | 1 | 1 | 1 |
| Yes | 2.16 (1.01, 4.59) | 2.72 (0.99, 7.51) | 1.80 (0.19, 17.53) | 7.27 (1.47, 35.87) * | 13.27 (2.44, 72.19) ** |
| **Place of child birth** |  |  |  |  |  |
| Elsewhere | 1 | 1 | 1 | 1 |  |
| Health facilities | 0.68 (0.11, 4.14) | 0.74 (0.08, 7.16) | 0.03 (0.00, 2.20) | 2.54 (0.22, 28.68) |  |
| **Antenatal visits** |  |  |  |  |  |
| <4 ANC visits | 1 | 1 | 1 |  | 1 |
| >=4 ANC visits | 1.17 (0.41, 3.38) | 0.70 (0.20, 2.46) | 0.24 (0.02, 3.08) |  | 1.64 (0.22, 12.30) |
| **Sociodemographic and household characteristics** | | | |  |  |
| **Maternal age (years)** |  |  |  |  |  |
| <24 | 1 | 1 | 1 |  |  |
| 25-34 | 2.29 (1.14, 4.60) * | 2.92 (1.05, 8.14) * | 7.36 (0.40, 135.09) |  |  |
| 35-49 | 3.41 (0.58, 20.07) | 3.97 (0.67, 23.47) | 60.63 (1.07, 3433.67) ** |  |  |
| **Caste/Ethnicity** |  |  |  |  |  |
| Brahmin/Chhetri | 1.02 (0.46, 2.27) | 1.02 (0.32, 3.27) | 3.07 (0.57, 16.54) |  |  |
| Madheshi | 0.87 (0.22, 3.52) | 0.40 (0.07, 2.41) | 5.70 (0.20, 163.11) |  |  |
| Dalit | 1.77 (0.55, 65.70) | 1.99 (0.46, 8.65) | 11.32 (0.39, 329.09) |  |  |
| Janajati | 1 | 1 | 1 |  |  |
| Muslim | 2.51 (0.49, 12.87) | 0.60 (0.03, 13.38) | 2.61 (0.06, 120.12) |  |  |
| **Maternal employment status** |  |  |  |  |  |
| Currently not working |  |  |  |  |  |
| Currently Working |  |  |  |  |  |
| **Wealth index** |  |  |  |  |  |
| Poorest | 1 | 1 | 1 |  |  |
| Poorer | 0.42 (0.09, 1.98) | 0.14 (0.02, 1.29) | 0.90 (0.12, 6.66) |  |  |
| Middle | **0.17 (0.03, 0.84) *** | 0.20 (0.02, 1.59) |  |  |  |
| Richer | 1.63 (0.51, 5.25) | 2.08 (0.38, 11.29) | 1.31 (0.07, 25.91) |  |  |
| Richest | 2.13 (0.55, 8.30) | 2.56 (0.34, 19.50) | 2.62 (0.14, 50.13) |  |  |
| **Media exposure** |  |  |  |  |  |
| Not at all | 1 | 1 | 1 |  |  |
| Less than once a week | 0.57 (0.21, 1.52) | 0.50 (0.16, 1.56) | 2.22 (0.03, 182.20) |  |  |
| At least once a week | 0.53 (0.22, 1.30) | 0.40 (0.13, 1.25) | 4.24 (0.03, 530.42) |  |  |
| **Household size (members)** |  |  |  |  |  |
| 1-3 | 2.43 (0.91, 6.54) | 2.95 (0.56, 15.48) | 1.02 (0.12, 8.73) |  |  |
| 4-5 | 0.84 (0.38, 1.88) | 0.79 (0.26, 2.38) | 0.19 (0.01, 2.97) |  |  |
| 6-38 | 1 | 1 | 1 |  |  |
| **Maternal education** |  |  |  |  |  |
| No education | 1 | 1 | 1 |  |  |
| Primary | 1.39 (0.24, 8.10) | 0.72 (0.09, 5.51) | 0.24 (0.02, 2.43) |  |  |
| Secondary and higher | 12.48 (2.63, 59.14) ** | 7.12 (1.07, 47.46) |  |  |  |
| **Paternal education** |  |  |  |  |  |
| No education | 1 | 1 | 1 | 1 |  |
| Primary | 0.69 (0.11, 4.38) | 0.75 (0.11, 5.14) | 1.24 (0.34, 4.48) | 0.78 (0.14, 4.44) |  |
| Secondary and higher | 0.96 (0.14, 6.48) | 0.93 (0.09, 9.98) |  | 0.54 (0.03, 9.21) |  |

***Significant at p-value < 0.001. **Significant at p-value < 0.01. *Significant at p-value < 0.05; 1.00 represents the reference category.
