# Supplementary material for: Commercial milk formula feeding among children under two years in Nepal: Trends and determinants from four Nepal Demographic and Health Surveys (2006–2022)
Source: PLoS One. 2026 Jan 2;21(1):e0339128. doi: 10.1371/journal.pone.0339128 (PMC12758697; doi:10.1371/journal.pone.0339128)
Supplement: S4 Table — Shows robustness checks using individual survey subsets for children aged 6–23 months. (DOCX) [file pone.0339128.s004.docx]

**S4 Table. Sensitivity analysis of the pooled analysis on subsets of data (NDHS 2022, 2016, 2011, & 2006) among children aged 6–23 months**

| **Variables** | **Pooled (2006-2022)** | **2022** | **2016** | **2011** | **2006** |
| --- | --- | --- | --- | --- | --- |
|  | **adjusted OR (95% CI)** | **adjusted OR (95% CI)** | **adjusted OR (95% CI)** | **adjusted OR (95% CI)** | **adjusted OR (95% CI)** |
| **Survey year** |  |  |  |  |  |
| NDHS 2006 | 1 |  |  |  |  |
| NDHS 2011 | 0.86 (0.39, 1.90) |  |  |  |  |
| NDHS 2016 | 0.60 (0.26, 1.41) |  |  |  |  |
| NDHS 2022 | 1.68 (0.80, 3.66) |  |  |  |  |
| **Enabling Factors** |  |  |  |  |  |
| **Place of residence** |  |  |  |  |  |
| Urban | 0.94 (0.61, 1.44) | 0.67 (0.34, 1.29) | 1.50 (0.71, 3.16) | 1.50 (0.58, 3.91) | 1.32 (0.26, 6.59) |
| Rural | 1 | 1 | 1 | 1 | 1 |
| **Province** |  |  |  |  |  |
| Koshi | 1 | 1 | 1 | 1 | 1 |
| Madhesh | 1.20 (0.55, 2.65) | 0.91 (0.28, 2.94) | 1.60 (0.27, 9.35) | 2.99 (0.26, 34.24) | 0.15 (0.00, 5.62) |
| Bagmati | 1.75 (0.99, 3.11) | 1.19 (0.47, 2.99) | 1.41 (0.44, 4.48) | 5.61 (1.81, 17.39) | 1.78 (0.37, 8.52) |
| Gandaki | 1.11 (0.52, 2.34) | 0.48 (0.08, 3.06) | 2.11 (0.67, 6.68) | 2.74 (0.63, 11.86) |  |
| Lumbini | 1.04 (0.54, 2.00) | 0.86 (0.30, 2.47) | 0.71 (0.19, 2.70) | 0.99 (0.20, 5.01) | 1.58 (0.32, 7.84) |
| Karnali | 1.10 (0.49, 2.46) | 0.70 (0.23, 2.12) | 1.76 (0.44, 6.95) |  | 2.03 (0.32, 12.69) |
| Sudurpaschim | 1.34 (0.57, 3.11) | 0.87 (0.29, 2.65) | 0.40 (0.07, 2.32) | 1.48 (0.28, 7.81) | 2.43 (0.59, 9.97) |
| **Underlying factors** | | | | |  |
| **Infant characteristics** |  |  |  |  |  |
| **Child sex** |  |  |  |  |  |
| Male | 1.67 (1.15, 2.42) ** | 1.68 (0.95, 2.97) | 1.22 (0.63, 2.39) | 2.52 (1.01, 6.28) * | 2.07 (0.79, 5.42) |
| Female | 1 | 1 | 1 | 1 | 1 |
| **Perceived size at birth** |  |  |  |  |  |
| Small | 2.76 ( 1.48, 5.15) ** | 2.83 (0.94, 8.52) | 0.78 (0.25, 2.47) | 3.75 (0.51, 27.33) | 3.27 (0.75, 14.31) |
| Average | 1.31 (0.74, 2.32) | 1.63 (0.66, 4.03) | 0.61 (0.22, 1.71) | 1.30 (0.22, 7.58) | 0.84 (0.23, 3.04) |
| Large | 1 | 1 | 1 | 1 | 1 |
| **Preceding birth interval** |  |  |  |  |  |
| No previous birth | 2.59 (1.02, 6.56) * | 1.86 (0.51, 6.82) | 2.37 (1.10, 5.11) * | 0.98 (0.34, 2.79) | 0.52 (0.09, 3.12) |
| <24 months | 1 | 1 | 1 | 1 | 1 |
| >=24 months | 2.51 (0.99, 6.37) | 2.06 (0.56, 7.58) |  |  | 1.29 (0.21, 7.80) |
| **Initiation of breastfeeding** |  |  |  |  |  |
| More than 1 hour |  |  |  |  |  |
| Immediately |  |  |  |  |  |
| **Obstetric and health service related characteristics** | | | | |  |
| **Provider of Delivery During Labour** |  |  |  |  |  |
| Health personnel | 1 | 1 | 1 | 1 | 1 |
| TBA/Relative/Others | 1.45 (0.67, 3.11) | 0.96 (0.37, 2.48) | 1.13 (0.29, 4.51) | 0.85 (0.07, 10.50) | 88.96 (13.37, 591.87) |
| No One | 1.25 (0.20, 7.87) |  | 0.30 (0.01, 6.08) | 7.61 (0.17, 337.62) |  |
| **PNC check within two days** |  |  |  |  |  |
| No | 1 | 1 | 1 |  | 1 |
| Yes | 1.26 (0.73, 2.18) | 1.29 (0.58, 2.88) | 1.56 (0.53, 4.60) |  | 1.50 (0.38, 5.93) |
| **Delivery by caesarean section** |  |  |  |  |  |
| No | 1 | 1 | 1 | 1 | 1 |
| Yes | 1.36 (0.90, 2.06) | 1.59 (0.84, 3.02) | 1.4 (0.63, 3.10) | 1.35 (0.45, 4.03) | 0.29 (0.04, 2.21) |
| **Place of child birth** |  |  |  |  |  |
| Elsewhere | 1 | 1 | 1 | 1 | 1 |
| Health facilities | 3.18 (1.16, 8.73) * | 1.97 (0.57, 6.73) | 1.49 (0.26, 8.47) | 2.30 (0.11, 48.04) | 130.56 (2.10, 1408.18) *** |
| **Antenatal visits** |  |  |  |  |  |
| <4 ANC visits |  |  |  |  |  |
| >=4 ANC visits |  |  |  |  |  |
| **Sociodemographic and household characteristics** | | | | |  |
| **Maternal age (years)** |  |  |  |  |  |
| <24 | 1 | 1 | 1 | 1 | 1 |
| 25-34 | 1.29 (0.89, 1.86) | 1.33 (0.70, 2.50) | 1.75 (0.78, 3.95) | 1.95 (0.74, 5.10) | 0.94 (0.31, 2.88) |
| 35-49 | 1.04 (0.43, 2.53) | 0.44 (0.10, 1.93) | 5.28 (1.52, 18.28) ** | 3.25 (0.23, 46.36) |  |
| **Caste/Ethnicity** |  |  |  |  |  |
| Brahmin/Chhetri | 1.01 (0.64, 1.59) | 1.79 (0.90, 3.55) | 0.59 (0.23, 1.52) | 0.38 (0.15, 0.98) * | 6.35 (2.11, 19.05) ** |
| Madheshi | 1.05 (0.54, 2.04) | 1.76 (0.65, 4.77) | 0.88 (0.16, 4.89) | 0.07 (0.01, 0.43) ** | 12.16 (2.32, 63.70) ** |
| Dalit | 0.69 (0.34, 1.37) | 1.88 (0.79, 4.48) | 0.11 (0.01, 0.92) * |  |  |
| Janajati | 1 | 1 | 1 | 1 | 1 |
| Muslim | 0.90 (0.30, 2.71) | 0.35 (0.04, 2.89) | 4.71 (1.01, 21.99) * |  | 32.58 (0.82, 1300.29) |
| **Maternal employment status** |  |  |  |  |  |
| Currently not working | 1 | 1 | 1 | 1 | 1 |
| Currently Working | 1.03 (0.69, 1.54) | 0.88 (0.46, 1.69) | 1.87 (0.95, 3.70) | 0.60 (0.24, 1.50) | 1.24 (0.46, 3.32) |
| **Wealth index** |  |  |  |  |  |
| Poorest | 1 | 1 | 1 | 1 | 1 |
| Poorer | 3.08 (1.35, 7.01) ** | 2.86 (1.06, 7.72) * | 0.72 (0.14, 3.82) | 0.31 (0.03, 3.24) | 0.60 (0.15, 2.49) |
| Middle | 3.02 (1.40, 6.51) ** | 2.43 (0.80, 7.43) | 2.87 (0.72, 11.46) | 0.72 (0.10, 5.00) | 0.20 (0.02, 1.95) |
| Richer | 3.01 (1.31, 6.92)** | 1.23 (0.36, 4.27) | 2.05 (0.49, 8.53) | 2.52 (1.01, 6.31) | 0.56 (0.16, 1.91) |
| Richest | 5.87 (2.42, 14.22)*** | 4.88 (1.31, 18.16) | 3.98 (0.69, 22.85) |  |  |
| **Media exposure** |  |  |  |  |  |
| Not at all | 1 | 1 | 1 | 1 |  |
| Less than once a week | 0.76 (0.42, 1.39) | 0.61 (0.28, 1.34) | 1.61 (0.38, 6.89) | 1.84 (0.46, 7.27) | 1.94 (0.35, 10.82) |
| At least once a week | 1.01 (0.59, 1.73) | 0.91 (0.46, 1.81) | 1.18 (0.27, 5.11) |  | 2.50 (0.41, 15.31) |
| **Household size (members)** |  |  |  |  |  |
| 1-3 |  |  |  |  |  |
| 4-5 |  |  |  |  |  |
| 6-38 |  |  |  |  |  |
| **Maternal education** |  |  |  |  |  |
| No education | 1 | 1 | 1 | 1 | 1 |
| Primary | 0.79 (0.38, 1.65) | 0.35 (0.13, 0.94) | 2.77 (0.76, 10.02) | 0.12 (0.01, 1.20) | 6.47 (1.29, 32.56) * |
| Secondary and higher | 1.50 (0.73, 3.08) | 0.94 (0.37, 2.39) | 2.24 (0.54, 9.40) | 1.79 (0.44, 7.27) | 7.81 (1.36, 44.89) * |
| **Paternal education** |  |  |  |  |  |
| No education | 1 | 1 | 1 | 1 | 1 |
| Primary | 2.22 (0.93, 5.33) | 2.50 (0.65, 9.55) | 9.13 (0.97, 85.93) | 0.62 (0.24, 1.58) | 0.39 (0.09, 1.77) |
| Secondary and higher | 3.14 (1.22, 8.10) * | 4.92 (0.99, 24.55) | 9.40 (0.88, 100.06) |  | 0.41 (0.08, 2.09) |

***Significant at p-value < 0.001. **Significant at p-value < 0.01. *Significant at p-value < 0.05; 1.00 represents the reference category.
