# Supplementary material for: Commercial milk formula feeding among children under two years in Nepal: Trends and determinants from four Nepal Demographic and Health Surveys (2006–2022)
Source: PLoS One. 2026 Jan 2;21(1):e0339128. doi: 10.1371/journal.pone.0339128 (PMC12758697; doi:10.1371/journal.pone.0339128)
Supplement: Attachment — Submitted filename: comments.pdf [file pone.0339128.s005.pdf]

Dear editor,

The manuscript significantly advances understanding of trends in commercial infant formula use in a low-income setting, with important implications for public health policy. While well-structured, scientifically rigorous, and highly relevant to global health priorities, its originality could be enhanced by drawing comparisons with other LMICs or proposing innovative intervention strategies.

Comments:

- To improve readability and professionalism, a thorough linguistic review is necessary.

Abstract:

- Condense repetitive phrases and focus on the most important findings (e.g. fewer mentions of "commercial formula").

Introduction:

- Briefly describe Nepal's unique socio-economic and policy context with regard to breastfeeding and formula feeding.

Method:

- Provide a brief discussion of potential bias (social desirability or recall bias) and how it was reduced.

- Some of the things that were included in the analysis (for example, how well the mother was educated, and what job the father did) could be explained more to make the idea behind the analysis clearer.

Results:

- Clearly state whether subgroup trends are crude (unadjusted) or adjusted for other covariates. If trends are unadjusted, it's important to qualify this limitation and emphasise the adjusted results from multivariable models in the discussion.

- Include explicit statistical comparisons, such as tests of trend significance over time (e.g. Cochran-Armitage or Mantel-Haenszel tests) and subgroup differences (e.g. interaction terms in regression models).

- Link the results more directly to public health priorities. For example, explain how an OR of 12.48 for maternal education translates into specific policy interventions, such as targeting educated mothers with breastfeeding awareness campaigns.

- The study does not address whether the results are robust to variations in assumptions or model specifications. For example:

- Were alternative age categorizations (e.g., 0-6 months instead of 0-5 months) tested?
- Were findings consistent across different years or subsets of data?

Include sensitivity analyses to demonstrate the robustness of the findings.

- The manuscript identifies disparities across provinces but does not delve into underlying reasons (e.g., why Bagmati province has the highest formula use or why rural areas have lower prevalence).

Hypothesise potential drivers of these differences (e.g. urbanisation, marketing exposure, cultural norms) and suggest how these findings could inform targeted interventions.

-Tables & figures: Add titles or legends to clarify abbreviations and statistical measures.

Discussion:

- Expand actionable recommendations, especially targeting maternal education and urban areas where formula feeding is prevalent.
