# Supplementary material for: Commercial milk formula feeding among children under two years in Nepal: Trends and determinants from four Nepal Demographic and Health Surveys (2006–2022)
Source: PLoS One. 2026 Jan 2;21(1):e0339128. doi: 10.1371/journal.pone.0339128 (PMC12758697; doi:10.1371/journal.pone.0339128)
Supplement: Attachment — Submitted filename: Reviewer comment_Infant formula paper_27.7.2025-Final.docx [file pone.0339128.s007.docx]

**Responses to Questions**

**Comments to the Author**

1. Is the manuscript technically sound, and do the data support the conclusions?

The manuscript must describe a technically sound piece of scientific research with data that supports the conclusions. Experiments must have been conducted rigorously, with appropriate controls, replication, and sample sizes. The conclusions must be drawn appropriately based on the data presented.

Reviewer #1: Partly

Reviewer #2: Yes

2. Has the statistical analysis been performed appropriately and rigorously?

Reviewer #1: No

Reviewer #2: No

3. Have the authors made all data underlying the findings in their manuscript fully available?

The [PLOS Data policy](http://track.editorialmanager.com/CL0/http:%2F%2Fwww.plosone.org%2Fstatic%2Fpolicies.action%23sharing/1/010f0196f91f3ba5-b0f2c303-9dc7-4474-8eff-630cfe2375af-000000/JooXrTaPov6tgJv9Wpfelevf64kslqf5Kn05leyeq5o=212) requires authors to make all data underlying the findings described in their manuscript fully available without restriction, with rare exceptions (please refer to the Data Availability Statement in the manuscript PDF file). The data should be provided as part of the manuscript or its supporting information, or deposited in a public repository. For example, in addition to summary statistics, the data points behind means, medians, and variance measures should be available. If there are restrictions on publicly sharing data—e.g. participant privacy or use of data from a third party—those must be specified.

Reviewer #1: Yes

Reviewer #2: Yes

4. Is the manuscript presented in an intelligible fashion and written in standard English?

PLOS ONE does not copyedit accepted manuscripts, so the language in submitted articles must be clear, correct, and unambiguous. Any typographical or grammatical errors should be corrected at revision, so please note any specific errors here.

Reviewer #1: No

Reviewer #2: Yes

5. Review Comments to the Author

Please use the space provided to explain your answers to the questions above. You may also include additional comments for the author, including concerns about dual publication, research ethics, or publication ethics. (Please upload your review as an attachment if it exceeds 20,000 characters)

Reviewer #1: Dear editor,
The manuscript significantly advances understanding of trends in commercial infant formula use in a low-income setting, with important implications for public health policy. While well-structured, scientifically rigorous, and highly relevant to global health priorities, its originality could be enhanced by drawing comparisons with other LMICs or proposing innovative intervention strategies.

**Author response**: Thank you for the feedback. We would like to draw your attention that there is no duplicity of publication using the same dataset. The outcome variable included in our work has never been reported from the dataset elsewhere.

**General Comment :**
1. To improve readability and professionalism, a thorough linguistic review is necessary.
**Abstract:**

**Author Response:** Thanks. A thorough linguistic review has been done, and changes have been made accordingly, which are reflected in the tracked changes version.

2. Condense repetitive phrases and focus on the most important findings (e.g., fewer mentions of "commercial formula").

**Author Response:** Thanks, now we have replaced it with the abbreviation CMF instead of Commercial Milk Formula, and have also used “formula milk” interchangeably based on the available literature.

**Introduction:**
3. Briefly describe Nepal's unique socio-economic and policy context with regard to breastfeeding and formula feeding.

**Author Response:** Thank you, now we have elaborated Nepal’s socioeconomic and policy context with regard to breastfeeding and formula feeding in the context section of the method. (Page:6 Paragraph: 2 ; Line ; 74 to 82 – clean version)

**Method:**
4. Provide a brief discussion of potential bias (social desirability or recall bias) and how it was reduced.

**Author Response:** Thanks a lot for the query about the way to minimise the potential bias, such as social desirability or recall bias. We have included additional information to explain about potential biases and the ways they were mitigated (where possible). Our paragraph reads:

*Finally, the IYCF data for 2006-2022 were gathered using the same core questionnaires, and the same institutions conducted the surveys. This consistency led to the accuracy and reliability of the estimates (26, 34-36). However, this study has some important limitations. The 24-hour recall for reporting formula-feeding practices may not accurately represent an individual’s usual formula-feeding practices or changes in feeding practices over time, which may underestimate actual use, as it excludes children who were fed formula outside of that timeframe. Also, this study could not include certain potential covariates important for predicting formula feeding practices, such as maternal knowledge and attitudes toward formula milk, as well as perceived insufficient breast milk, which was considered in previous studies (67). Including these variables could affect the study findings. (Page 16; Paragraph 1)*

**5. Some of the things that were included in the analysis (for example, how well the mother was educated, and what job the father did) could be explained more to make the idea behind the analysis clearer.**

**Author Response:** Thanks a lot for the comment. Now, mother’s education and father’s occupation have been explained further in the method section regarding their influence on formula feeding practices. Please check the line number : (Page number 9, Paragraph 1, Line number 181-191, clean version). Additionally, we have also provided an additional file to explain how each exploratory variable, including the two suggested by the reviewer (Supplementary Table 1).

*“In addition, mother’s education and father’s occupation were included as key determinants of CMF feeding based on theoretical and empirical evidence. Mother’s education influences the knowledge, attitudes, and decision-making practices related to child nutrition. Higher levels of education have been associated with both increased awareness of breastfeeding benefits and, paradoxically, a greater likelihood of CMF use, particularly among mothers from urban and wealthier communities (6, 45). Likewise, a father’s occupation may determine the family’s socioeconomic status, time availability, and support for breastfeeding practices. Furthermore, the father’s crucial role in financial decision-making can shape the accessibility and affordability of CMF, especially in contexts where fathers have higher control over the household resources (46)” (Page 9; Paragraph 1; Line: 183-192 : Clean version)*

**Results:**
6. Clearly state whether subgroup trends are crude (unadjusted) or adjusted for other covariates. If trends are unadjusted, it's important to qualify this limitation and emphasise the adjusted results from multivariable models in the discussion.

**Author Response:** Thank you for your comment. The subgroup trend presented in our manuscript is crude (unadjusted). Now we have corrected in our result section, and reported it as a limitation in our discussion section. Please check page 16, para 1, Line 412-414: clean version.

Meanwhile, we have emphasised the adjusted results from multivariable models in the discussion. Please check page 12, lines 268-288: clean version

7. Include explicit statistical comparisons, such as tests of trend significance over time (e.g., Cochran-Armitage or Mantel-Haenszel tests) and subgroup differences (e.g., interaction terms in regression models).

**Author Response:** We have presented the trend of CMF feeding practices from 2006-2022, stratified by different variables of interest (province, education etc). In this regard, we have simply plotted the prevalence of CMF feeding according to the variable of interest, and have not assessed the trend as significant or not, as it was not the objective of our study. Regarding subgroup differences, we did not have any predefined specific exposure variable of interest, with which we wished to show the association of commercial milk formula. That’s why we didn’t have any predefined hypotheses regarding interaction, and interaction terms were not included in the regression model guided by our study objectives.

8. Link the results more directly to public health priorities. For example, explain how an OR of 12.48 for maternal education translates into specific policy interventions, such as targeting educated mothers with breastfeeding awareness campaigns.

**Author response:** Thanks a lot for the insightful comment. We have reported and discussed it in our discussion section rigorously. Please refer to: page 14; Para 2; Line number 349-365: Clean version.

“Our study reported that higher parental education is associated with increased use of CMF. This finding reflects the complex interplay between education, employment, and childcare practices. Increased female education in LMICs is associated with higher labour force participation (54). Returning to work is linked to poorer breastfeeding practices in the absence of a supportive environment for continuous breastfeeding (55). One crucial factor contributing to this, particularly for working mothers, is the duration and accessibility of maternity leave. In Nepal, maternity leave is officially three months, primarily for women with permanent jobs in the government sector; private and unorganized sectors often lack such provisions. This limited leave can compel mothers to introduce CMF, creating a significant occupational and family/child conflict that impedes optimal breastfeeding. Therefore, a critical policy implication is the urgent need for universalizing and enforcing comprehensive paid maternity leave across all employment sectors in Nepal, alongside initiatives to create breastfeeding-friendly workplaces that include accessible lactation facilities and flexible work arrangements. Similar trends have been observed in other countries. A significant negative association between mothers’ education and EBF practices was reported in Pakistan, China, Saudi Arabia, the United Arab Emirates, and Lebanon (56-60)^.^”.

9. The study does not address whether the results are robust to variations in assumptions or model specifications. For example:

9.1 Were alternative age categorizations (e.g., 0-6 months instead of 0-5 months) tested?

**Author response:** We used the standard 0–5 month categorization in line with WHO recommendations for exclusive breastfeeding, which is an inclusive class interval and takes into account completed months of age.

9.2. Were findings consistent across different years or subsets of data?
Include sensitivity analyses to demonstrate the robustness of the findings.

**Author Response**: Thanks a lot for the comment. We pooled the data from four DHS waves (2006, 2011, 2016, and 2022) to increase the statistical power and improve the precision of the estimates for subgroup analysis. Hence, we didn’t include a sensitivity analysis to demonstrate the robustness of the findings. However, as per your suggestion, we have performed the sensitivity analysis and have reported it in our supplementary **tables S3 and S4**.

10. The manuscript identifies disparities across provinces but does not delve into underlying reasons (e.g., why Bagmati province has the highest formula use or why rural areas have lower prevalence). Hypothesise potential drivers of these differences (e.g., urbanisation, marketing exposure, cultural norms) and suggest how these findings could inform targeted interventions.

**Author Response:** Thanks a lot for the insightful comment. We have included the possible reasons for regional disparities in our discussion section. These include some of the factors mentioned by the reviewer.

*”Our analysis reveals significant disparities in CMF feeding across provinces in Nepal. Infants aged 0-5 months in Karnali and Sudurpaschim provinces are less likely to be CMF-fed than those in Koshi province. Karnali and Sudur-Paschim provinces have the lowest urban population compared to the national urban population (52). Our findings, therefore, align with studies documenting the relationship between urbanization and CMF both within the Nepalese context (23) and in the broader literature (12,13,22). Specifically, the higher prevalence of CMF in more urbanized areas of Nepal, like Koshi province, is consistent with evidence from other regions. Baker et al. and Robes et al (12,13) highlight how the proliferation of corporate marketing of CMF is particularly effective in changing feeding environments in urban settings within low- and middle-income countries. ’ (Page 13; Line 325-334: Clean version )*

We acknowledge the importance of these factors. However, due to a lack of a qualitative component, we can not ascertain if and how much these factors influence the varied commercial formula use in the regions.

14. Tables & figures: Add titles or legends to clarify abbreviations and statistical measures.

**Author response:** Thank you for this helpful suggestion. We have carefully reviewed all tables and figures and have added appropriate titles and legends to clarify abbreviations, statistical measures, and model outputs. These revisions aim to improve readability and ensure clarity for readers who may be less familiar with the terminology used.

**Discussion:**
15. Expand actionable recommendations, especially targeting maternal education and urban areas where formula feeding is prevalent.

**Author response**: Thank you for your valuable feedback. We have already addressed your comment regarding policy implications by integrating them into the relevant areas of the discussion/conclusion section in each paragraph, as well as refining the conclusion to reflect these broader implications. Please check Page number 14, 16; Line number 346-348; 355-362; 391-394; 428-436 (Clean version)

**Reviewer #2: Trends in and determinants of commercial milk formula feeding practices among children aged 0-23 months in Nepal: Pooled Analysis of four Nepal Demographic and Health Surveys (2006 - 2022)**

(PONE-D-24-48446)

Dear editor,

Thank you for inviting me to review the manuscript “Trends in and determinants of commercial milk formula feeding practices among children aged 0-23 months in Nepal: Pooled Analysis of four Nepal Demographic and Health Surveys (2006 - 2022)”. The study is informative, and it has the potential to provide valuable insights for policymakers to ensure good health for infants in Sub-Saharan Africa. However, some points need to be addressed before the study can be accepted for publication.


**Comments**

**Introduction:**

1. “….However, less than half (48%) of 0-5 months infants were exclusively breastfed globally 24 hours before the surveys. Does it mean that in all 4 surveys, 48% of infants were exclusively fed, respectively? Kindly revise the statement for clarity.

**Author response**: Thank you for seeking clarity. We have presented it as a global prevalence based on UNICEF-provided figures, which they update periodically based on different survey estimates.

Please revise the following sentence for clarity. “Only one study has directly examined the commercial milk formula used in Nepal, limited to a specific region.”

**Author response**: Thank you. Now we have revised this sentence. Please check Page 5, para 2, line numbers 55-57 (Clean version).

“Only one study has directly examined the CMF use in Nepal, limited to a western region.”

2. Regarding the dependent variable, indicate the exact question that was asked in the NDHS datasets and the original coding.

**Author response:** Thanks a lot for highlighting the need for clarity on the measurement of the outcome variable of interest, which is formula feeding in our study.

“In NDHS: Mothers of children aged 0–23 months were asked in the Women’s Questionnaire whether their child had consumed infant formula such as Lactogen, Farex, or Nan in the previous 24 hours, alongside items like breastmilk, animal milk, and other liquids. Now we have edited it in Page number 8; Para 1; line number 142-148 (Clean version) of our manuscript.”

**Methods:**

3. As part of the context, let readers know about Nepal as a country regarding its location, population size, and other socio-demographic characteristics.

**Author response:** Thanks a lot for the suggestion. Now we have briefly elaborated on Nepal as part of the context relevant to breastfeeding. Kindly check the page no 6, Para 2; line numbers 74-82 (Clean version)

4. Indicate the benefit of using nationally representative data

**Author response:** Thanks a lot for the comment. We have reported the benefit of using nationally representative data as a strength and limitation paragraph under the discussion section – strengths and limitations. Kindly check Page number 15/16; Line number 395-397 (Clean version).

5. The measurement of feeding a baby with formula 24 hours before the survey seems problematic, as it cuts off those who were not fed within that period. People may be using the formula, but may not have used it within the 24 hours. This should be captured as a limitation.

**Author response:** Thanks a lot for suggesting to keep the recall period in the limitation. We have kept it in the limitations section. Kindly check the Page number 16; para 1; line number 409-412).

6. Give a brief description of the ecological model before describing your variables with it

**Author response:** Thanks. Now we have provided a brief description of the ecological model, which has been adapted in our study to keep the variable under two domains, i.e., enabling and underlying factors, which influence the formula feeding practices directly and indirectly, respectively. Please check the Page number 8; Para 2; Line number 153-160 (Clean version).

7. Give some examples of the several household items used to measure wealth status. It’s also better to maintain wealth status than SES.

**Author response:** Thanks a lot for the insightful comment. Now we have used wealth status instead of socioeconomic status, and also have reported the list of household items used to measure the wealth status in the method section. Kindly check the Page number 9; Para 1; line number 177-183 (Clean version).

8. In your analysis, you stated that “…which used a two-stage technique based on a conceptual framework described by Victoria et al. (40)., What is the framework about? Please spell it out for clarity. Importantly, you have highlighted that the ecological model is guiding the study. In this case, one would expect that it drives the analysis, but that is missing.

**Author response:** Thank you for your comment. In this study, determinants of CMF feeding practices are sought. As Victoria et. al. complex hierarchical relationships between these determinants (enabling and underlying factors in our case) are best managed through the use of conceptual frameworks, and for this purpose we have adapted the ecological model to group our explanatory variables of interest at two stages.

9. Furthermore, looking at the nature of the model, it’s best to perform a multilevel modelling to ascertain the factors having the most impact. It will also cater for the complex nature of the sampling technique. Is there any reason for not employing that approach?

**Author response:** Thanks a lot for the critical comment, and we acknowledge this. However, we did not use multilevel modelling in this study because the DHS data were analysed using survey commands in Stata that account for the complex sampling design through stratification, clustering, and sample weights. While multilevel modelling can account for hierarchical data structures, it requires appropriate weights at each level. The DHS only provides weights at the household and cluster levels, and not at higher levels (e.g., region or province), which limits the accurate application of multilevel modelling in this context.

10. It’s better to first describe the socio-demographic characteristics of the respondents before other variables. This will provide contextual understanding of other subsequent findings

**Author response:** Thanks, now we have moved the sociodemographic characteristics ahead of the description of other variables. Please check our result section, Page 10; Para 3; Line: 218 onwards (Clean version).

11. The regression Table should be labeled 3 instead of 2 to distinguish it from the chi-square table, which is labeled 2

**Author response:** Thanks a lot for your suggestion. The regression table is labelled as 3, and the chi-square table is labelled as Table 2.

12. From the regression Table, it appears you performed two models, one for 0-5 and 6-23; however, this is not clear in the methodology. Although you mentioned the two-stage technique, this is not clear. Kindly clarify it.

**Author response: T**hank you for your helpful comment. We acknowledge the need to clarify the terminology used. In our analysis, we stratified the sample into two age groups—0–5 months and 6–23 months—to reflect differences in infant and young child feeding recommendations and practices across these age ranges. This age-based stratification allowed for a more nuanced analysis of formula feeding patterns, and it has been reported in the outcome variable, which we have mentioned. Please check Page 8; Para 1; line numbers 140-143.

The reference to “two-stage analysis” in our manuscript was not in relation to the age stratification but rather reflected our analytical approach based on the conceptual framework. Specifically, we sequentially introduced enabling and then underlying factors into the regression models to assess their independent and combined influence on formula feeding practices. We have now revised the Statistical Analyses section to clarify this distinction. Please check Page no 9; Para 2; Line number 199-202 (Clean version).

13. Please insert the figures of the variables that were not significant in the regression Table

**Author response:** We have included figures of the variables that were of important consideration. Due to having a large number of explanatory variables, we respectfully decline this suggestion. However, Table 2 provides information on the prevalence of CMF by various explanatory variables considered in this study.

**Discussion**

14. “Our findings align with the recent studies reporting a decreasing trend of EBF in Nepal from 2011 to 2022 NDHS”. Relating your findings to studies that used the same or a section of the NDHS is quite problematic. It is best to discuss your findings with other Asian countries.

**Author response:** We appreciate your constructive feedback. We have paraphrased and added references to evidence from neighbouring countries (India and Bangladesh). Please see below the revised para in Page 12/13, beginning from Para 5, Line number 297-306 (clean version)

*“Our analysis shows a substantial increase in CMF feeding among infants aged 0-5 months in Nepal between 2006 and 2022. Considering the significant consequences of CMF on human and planetary health (12,15,16), this trend is concerning, as it indicates a shift from the recommended EBF practice. This alarming trend also reflects global increases in commercial milk formula use, with global sales of CMF approaching US$55 billion annually by 2023 (2). Similar increases in commercial milk formula use and challenges to breastfeeding practices have been observed in other Asian countries. For instance, studies in India (48), and Bangladesh (49) have also reported rising trends in formula feeding. These regional parallels underscore the shared challenges in promoting optimal infant feeding practices across Asia.”*

15. One key reason that might contribute to the increase in the use of infant formula is occupational and family/child conflict. What’s the duration of maternity leave in Nepal? Don’t you think this might partly explain it? Any policy recommendation in that direction?

**Author response:** Thanks a lot for the comment. The duration of maternity leave is three months in Nepal, but only among those who have a permanent job in the Government sector, not in other sectors. Plus, businesses and the unorganised sector do not have those leaves in place, which can come as a challenge. We have reported it in the discussion section. Kindly check Page 14; Para 2; line numbers 353-360 (clean version).

16. “Our findings align with studies documenting the relationship between urbanization and commercial milk formula.” Kindly provide context.

**Author response:** Thank you for your constructive feedback. We have paraphrased Page 13, Para 3, Line number 328 – 339 (Clean version) to provide more contextual information as below:

“Our findings, therefore, align with studies documenting the relationship between urbanization and CMF both within the Nepalese context (23) and in the broader literature (12,13,22). Specifically, the higher prevalence of CMF in more urbanized areas of Nepal, like Koshi province, is consistent with evidence from other regions. Baker et al. and Robes et al (12,13) highlight how the proliferation of corporate marketing of CMF is particularly effective in changing feeding environments in urban settings within low- and middle-income countries. Urban areas often provide increased physical proximity to CMF products, greater exposure to their aggressive marketing, and may offer perceived solution to the challenges such as higher rates of maternal work outside of the home, shrinking family sizes (shift from larger kin-dominated to smaller nuclear units), and the adoption of ‘modernity’ values and lifestyles more conducive to formula-feeding. ”

17. Generally, the discussions lack policy implications. The author(s) need to discuss important policy implications related to the findings**.**

**Author response:** Thank you for your valuable feedback. We have addressed your comment regarding policy implications by integrating them into the relevant areas of the discussion section, as well as refining the conclusion to reflect these broader implications. Please check Page number 14, 16; Line number 346-348; 355-362; 391-394; 428-436 (Clean version)
