# Supplementary material for: Commercial milk formula feeding among children under two years in Nepal: Trends and determinants from four Nepal Demographic and Health Surveys (2006–2022)
Source: PLoS One. 2026 Jan 2;21(1):e0339128. doi: 10.1371/journal.pone.0339128 (PMC12758697; doi:10.1371/journal.pone.0339128)
Supplement: Attachment — Submitted filename: Formal point to point response_16.11.2025.docx [file pone.0339128.s008.docx]

# Response to Reviewer Comments

16/11/2025

To: The Editor
Subject: Response to Reviewer Comments – Manuscript Revision

Dear Editor,

Thank you for the opportunity to revise and resubmit our manuscript. We appreciate the constructive feedback provided by the reviewers, which has significantly improved the quality of our work. Below, we provide a detailed point-by-point response to all comments. While responding to reviewers, we have provided references to specific line numbers in our cleaned version of the manuscript.

## Reviewer #1 Comments and Responses

1. Comment: The title/topic is quite long. Kindly try to shorten it.

**Response:** We have revised the title to: “Commercial milk formula feeding among children under two in Nepal: Trends and determinants from four Nepal Demographic and Health Surveys (2006–2022)”.

2. . “…. However, less than half (48%) of 0-5 months infants were exclusively breastfed globally, 24 hours before the surveys. “Author response: Thank you for seeking clarity. We have presented it as a global prevalence based on UNICEF-provided figures, which they update periodically based on different survey estimates.” In this case, it would be better to state the year and mention UNICEF’s report in the statement to provide a clearer understanding of the survey you are referring to.

**Response:** Thanks. We have now mentioned the source of the updated breastfeeding estimates in the text. Please check line numbers 11-14, “However, according to the updated global estimates by UNICEF, less than half (48%) of infants aged 0-5 months were exclusively breastfed globally within the 24 hours preceding the surveys. Similarly, only 59% of children aged 12- 23 months continue breastfeeding for two years (4).”

3. Reference 4 has been repeated in the reference list. It appears you stated WHO but referenced UNICEF. Please recheck the entire list of references for corrections where necessary.

**Response:** Thank you for bringing your oversight to our attention. We have corrected the duplication and verified the reference list. Hence, we have replaced reference 4 with 5. Please check line numbers 14-17: “WHO and UNICEF expect all member states to achieve 70% EBF rates in the first six months of an infant’s life and 60% continuation rates of breastfeeding among children up to two years of age by the end of 2030 (5).”

4. Lines 32 to 34“…. this growth is expected to continue, with sales projected to rise by another 10.8%, reaching 2.38 million tons by 2024 (12), reflecting a situation where more infants and young children are provided.” We are currently in 2025, hence you cannot project to 2024. Kindly revise it.

**Response:** Thank you for the feedback. We have now updated the projected figure for CMF sales by 2030 and provided an appropriate citation. Please check lines 32-36.

“The global retail sales of CMF expanded by 121.5%, increasing from 0.97 to 2.15 million tonnes from 2005 to 2019 (12); this growth is expected to continue, with sales projected to rise by another 18.6 %, reaching 2.55 million tonnes by 2030 (13), reflecting a situation where more infants and young children are provided CMF than ever (14).”

5. Lines 158 to 164 seem incomplete. Recheck and complete it for easy understanding. Besides, the word firstly should be followed by secondly or an alternative phrase

**Response:** Thank you. We have now rewritten this section for clarity and presented it in a revised format. Please check line numbers 162-172

“This analysis included data from the four consecutive NDHS waves (2006, 2011, 2016, and 2022), with the survey year treated as a potential confounder. Variables were grouped into two broad categories: (1) enabling factors, including province (Koshi, Madhesh, Bagmati, Gandaki, Lumbini, Karnali, Sudurpaschim), place of residence (urban/rural), and ecological zone (mountain/hill/terai); and (2) underlying factors, including maternal characteristics (age, education, employment), paternal characteristics (education, employment), socioeconomic characteristics (caste/ethnicity, wealth index, household size, exposure to media), health service related characteristics (assistance during delivery, postnatal care within two days, caesarean section birth, place of childbirth, number of ANC visits), and child characteristics (age, sex, perceived size at birth, preceding birth interval, initiation of breastfeeding)”.

6. Line 198:” and respective chi-square tests (were employed” recheck the sentence. There is an omission from the bracket.

**Response:** Thanks. Now it has been corrected as per the suggestion. Please check lines 203-205.

“The distribution of formula feeding by participants’ background characteristics was presented as proportions, and respective chi-square tests χ^2^) were used to assess univariable associations.”

7.199-202: Specify the two-stage techniques you used. “Specifically, we sequentially introduced enabling and then underlying factors into the regression models to assess their independent and combined influence on formula feeding practices. We have now revised the Statistical Analyses section to clarify this distinction. Please check Page no 9; Para 2; Line number 199-202. Your response to my previous comment gives insight into the section, but it’s not captured in the manuscript.

**Response:** Thank you. This has now been reflected in our manuscript. We have revised the paragraph accordingly. Please check the lines 206-211 as: “A two-stage technique based on a conceptual framework described by Victoria et al. (49) was employed. This technique sequentially introduced enabling and then underlying factors into the regression models to assess their independent and combined influences on formula-feeding practices. Sensitivity analysis was conducted by performing separate regression analyses for each year to assess the consistency of findings over time.”

8. Lines 218 to 224 give some statistics in your description. Line 223: Insert a comma after ‘working women’ to make the sentence clearer.

**Response:** Thank you for the feedback. We have presented some statistics in this section of the results. Please see line number xx-xx

**“Table 1** presents the background characteristics of study participants, based on pooled data from the last four NDHS surveys (2006-2022). Most mother-child pairs (0-5 months and 6-23 months) were from rural settings (64.3%) and the Terai region (58.2%). Most of the children had a preceding birth interval of ≥ 24 months (50.8 %), and around one out of ten (10.1 %) were born through caesarean section. Regarding household and maternal characteristics, most mothers were ≤ 24 years old (56.5%), working women (58.6%), had media exposure at least once a week (46.9%), and had attained secondary education or higher (42.8%). We have also inserted a comma at the appropriate place to make the sentence clearer.

9. Line 231, delete in 2016 and 2022 after the respective

Response: Thank you. Now we have omitted. Please check the line numbers 236-238

“…but showed a significant increase in 2016 and 2022, reaching 4.52% (95% CI: 2.55, 7.87) and 11.09% (95% CI: 8.01, 15.16), respectively (**Figure 3A**)”

10. Give appropriate labeling of your figures. For example, Figure 1: Flow chart of sample size selection of participants. Besides, label each figure under the referred figure for easy identification. You have stated Figure 3, Figure 3A, and Figure 3B, but these cannot be found since they have been labeled as A and B under Figure 3. Kindly label each as indicated in the write-up.

**Response:** Thank you for identifying inconsistencies. We have relabeled all figures in accordance with the journal’s guidelines.

Figure 1. (Flow chart of sample size selection of study participants)

Figure 2. (Ecological model of enabling and underlying factors influencing formula feeding among children)

Figure 3. (Trends in commercial milk formula feeding practices among children (2006-2022)

11. Line 244: insert with after fed

**Response:** It has now been corrected. Please check line numbers 260-261

The proportion of infants (0-5 m) fed with CMF in ………….

13. Line 245 …. in 2011 but significantly increased to 30.64 %...” Which year are you referring to? 2022? Also, correct the sentence beginning with “while”

**Response:** Thank you for bringing the error to our attention. It has been resolved now. Please check line numbers 260-261

The proportion of infants (0-5 months) fed with CMF in the richest wealth quintile rose sharply from 11.81% in 2006 to 34.62% in 2022 (**Figure 4B**).

14. The Tables have still not been labeled well. As indicated earlier, the regression analysis is labeled 2, likewise the cross-tab Table.

**Response:** Thank you. Now, the regression table is labelled 3, while the cross-tab is labelled 2. Please check line numbers 271-275

**Table 2** presents the distribution of CMF feeding by background characteristics. The factors significant in the Chi-squared test at p < 0.005 were further were further analysed using logistic regression. **Table 3** presents the factors associated with formula feeding practices among children, stratified by age categories (0-5 months and 6-23 months) based on pooled data from 2006 to 2022.

15. Line 268, revise the sentence for understanding

**Response:** Thank you, that line has now been revised for clarity and understanding. Please check line numbers 277-279

Among infants aged **0-5 months**, those from the NDHS 2022 were 5.40 times more likely to be fed formula milk (adjusted odds ratio (aOR): 5.40; 95% confidence interval (CI): 1.41, 0.77) compared to those from the 2006 survey.

16. Lines 307-311 should be a continuous sentence

**Response:** Thank you. The line has been made continuous. Please check line numbers 315-319

Despite multiple government initiatives, including the National Nutrition Strategy (2004), the 2014 IYCF strategy, and the Multi-Sector Nutrition Plan-III (2023–2030), Nepal continues to face significant challenges (52). These findings have direct implications for achieving Nepal’s commitment to SDG and the WHO Nutrition Targets, which emphasize optimal breastfeeding practices as a cornerstone of child survival and development.

17. line 336, change ‘must’ to should

**Response:** Thanks. “Must” has been replaced with “should” within the text. Please check the lines 354-356.

Policymakers should strengthen regulatory bodies, impose stricter penalties for violations, and monitor online and indirect marketing tactics that circumvent traditional advertising bans.

18. Line 432: Be specific on the vulnerable groups or target population. Also, indicate the people to enforce the stated policies or interventions. Recommend also for future studies. For example, do you perceive that a qualitative analysis on the issue will be useful?

**Response:** Thank you. Now this section has been extensively edited, and necessary changes have been made accordingly. Please review lines 439-444.

Priorities include enforcing existing legislation against aggressive CMF marketing, enhancing breastfeeding support in health facilities, expanding paid maternity leave, and implementing comprehensive policies that address societal and occupational barriers to breastfeeding. Future studies should investigate the underlying drivers of formula feeding practices, particularly through qualitative research that examines cultural norms, maternal perceptions, and barriers to exclusive breastfeeding.

19. There are still typographical errors in the manuscript that need correction. I have indicated a few, but the authors need to work on the errors

**Response:** Thank you for identifying typographical errors, which have helped me rectify the mistakes made in the manuscript.

## Reviewer #3 Comments and Responses

This article is important for policymakers in the health sector. I believe urgent actions and steps are necessary to address this situation. I have some comments General comments
1. This article needs linguistic review and formatting editing.

Response: Thank you so much for your feedback. We appreciate your observation regarding the language and formatting of the manuscript. We have carefully revised and rerevised the text to improve clarity and overall readability.

Additionally, we have ensured that the formatting adheres to the journal’s guidelines.

2. There are some sentences that were used many times, so they must be abbreviated.

**Response:** Commonly repeated phrases have been abbreviated. For example, commercial milk formula and exclusive breastfeeding.

**Introduction**

3. Describe more information on the demography and geography of Nepal

**Response:** Thank you very much for the feedback. We have discussed information on Nepal’s demography and geography under the “Context/study setting” subsection within the Methods section. To keep the introduction concise, we have included the Nepalese context in the Methods section, which also helps avoid repetition.

4. Can you explain the reasons behind the illegal places of selling commercial milk formula in Nepal, even in Urban areas, where the government is supposed to have more authority and power? Is there a role for gangs, smugglers, foreign affairs, or the law?

**Response:** Thank you for your inquiry. The widespread sale of commercial milk formula in Nepal is largely due to the weak implementation of existing laws, although new laws are being introduced and amended. We have mentioned this in our introduction section. Please see line numbers 109-115

However, this act is currently under review and has been submitted to the Ministry of Law, Justice, and Parliamentary Affairs for approval pending tabling in parliament. Enforcement of this law has been poor and inconsistent, even after three decades since its endorsement (35). Formula milk is distributed nationwide through pharmacies, grocery stores, and department stores. Such outlets and sales in urban areas are common and often unregulated due to inadequate enforcement of the existing laws (23, 35).

5. You mention that less than half (48%) of 0-5-month-old infants were exclusively breastfed globally 24 hours before the surveys. Does it mean that it is really exclusive, BF?

**Response:** UNICEF regularly provides a global update of EBF based on reporting from different countries (provided by survey estimates such as MICS, DHS). This nationally representative survey collects the breastfeeding history from the mother of the infant within the past 24 hours. So, there is often a limitation on the definition of EBF reported by these survey estimates.

6. The commercial milk formulas that are present in the local markets in Nepal are locally produced or imported?

**Response:** Thank you for your query. CMF present in the local markets of Nepal is imported from other countries (mainly from India).

**Methods**
7. Context of the study, it is better to be shifted to the introduction section

**Response:** Thank you for your suggestion. We considered placing the study context in the introduction; however, doing so would make the introduction excessively lengthy and repetitive. Including the context/study setting within the methods section ensures clarity and maintains a concise structure, which aligns with common practices and the overall flow of the manuscript.

8. The role of recall bias

**Response:** Thank you for your feedback. We have reported the role of recall bias in our limitations section under discussion.

The NDHS and other DHS surveys worldwide use the 24-hour recall method to collect data on EBF. This is a pragmatic methodology and has been recommended by the WHO and UNICEF in the IYCF guideline as a feasible method to measure the prevalence of EBF (REF). We have indicated that EBF was based on a 24-hour recall in the revised section. (Line 414-417)

The limitation section reads:

First, the 24-hour recall method for reporting formula-feeding practices may not accurately represent an individual’s usual formula-feeding practices or changes over time, potentially underestimating actual use, as it excludes children who were fed formula outside of that timeframe.

9. Before using the ecological model to describe your variables, briefly describe it.

**Response:** Thank you. We have provided a brief description of the ecological model before describing the explanatory variables in the method section. Please find the line number 157-162

The ecological model is a theoretical framework used to understand the multiple levels of influence on individual behaviours, including interpersonal, organisational, community, and societal factors. It emphasises that health behaviours are shaped not only by individual-level determinants but also by the social and environmental contexts in which individuals live (42). Addressing these interconnected layers is essential for promoting optimal feeding behaviours.

**Results**

10. About 35% of the sample size resident in the urban areas, is it reflect real population distribution across the country?

**Response:** Thank you for your observation. We have used pooled data from 2006 to 2022. Between 2006 and 2022, Nepal experienced urbanisation and underwent changes to its administrative structure, resulting in a more urban population, according to the latest estimates. Hence, the reported figure of 35 % for the urban sample, based on pooled data, may not accurately reflect the current urban-rural population distribution in Nepal, which has been acknowledge under the limitation section. See line numbers 423-427

Lastly, Nepal underwent significant urbanisation between 2006 and 2022, accompanied by administrative restructuring after 2015, which contributed to an increase in the urban population according to recent estimates. Consequently, the reported urban figure of 35 % based on pooled data may not accurately represent the current urban-rural population scenario in Nepal.

11. Indicate clearly if subgroup trends have been adjusted for other factors or are crude (unadjusted). It's crucial to clarify this restriction and highlight the corrected outcomes from multivariable models in the discussion if trends are not adjusted.

**Response:** Thank you for your critical comment. We have presented the unadjusted trends of formula feeding in the subgroup analysis. Moreover, we have limited our discussion to adjusted analysis only. Subgroup trends are unadjusted; the discussion focuses on adjusted analysis. We have clarified this in our revised version, and the paragraph (Line number 421-424) reads:

Thirdly, trends presented in this study of formula feeding practices over the years were unadjusted estimates, which may partly reflect changes in underlying factors rather than true shifts in the outcome of interest.

**Discussion**
12. Make a stronger connection between the findings and public health priorities.

**Response:** Thanks for your valuable feedback. We appreciate the suggestion to strengthen the link between our findings and public health priorities. In our manuscript, we have linked public health priorities to important findings that address the high prevalence of formula feeding. We have extensively revised our discussion section and linked it with the recommendation (visible in the track-changed version). Please note that we have discussed policy recommendations and public health priorities in relation to each key finding in our study.

**References**
Follow the journal guideline regarding reference writing
13. There are some references, either very old or relatively old. So, it should be changed

**Response:** Thank you for bringing this to our attention. We appreciate the observation regarding the age of some references. We have carefully reviewed the reference list and updated older citations with more recent and relevant literature. This ensures that the manuscript reflects current evidence and aligns with the recent development in the field.
