# Supplementary material for: Commercial milk formula feeding among children under two years in Nepal: Trends and determinants from four Nepal Demographic and Health Surveys (2006–2022)
Source: PLoS One. 2026 Jan 2;21(1):e0339128. doi: 10.1371/journal.pone.0339128 (PMC12758697; doi:10.1371/journal.pone.0339128)
Supplement: Attachment — Submitted filename: Point by point response_28.11.2025.docx [file pone.0339128.s009.docx]

# Response to Editor Comments

28/11/2025

To: The Editor
Subject: Response to Reviewer Comments – Minor correction in supplementary table

Dear Editor,

Thank you for the opportunity to revise our supplementary table. We appreciate the constructive feedback you provided. Below, we provide a detailed point-by-point response to a couple of comments.

The authors have satisfactorily responded to all reviewers’ comments. However, there are two minor corrections to be made below:

1. In Supplementary Table S3, there is an issue with the household size categories: they are presented as 1-Mar, 4-Mya, and Jun-38. Please correct the categories to 1-3, 4-5, and 6-38.

Response: Thank you for your comment. We have now edited our supplementary table S3 and uploaded the corrected version.

2. Supplementary Table S4 has the same issue regarding the household size categories: they are presented as 1-Mar, 4-Mya, and Jun-38. Please correct the categories to 1-3, 4-5, and 6-38.

Response: Thank you for your observation. We have now edited our supplementary table S3 and uploaded the corrected version.
